# Experiences of Latinos with limited English proficiency with patient registration systems and their interactions with clinic front office staff: an exploratory study to inform community-based translational research in North Carolina

**DOI:** 10.1186/s12913-015-1235-z

**Published:** 2015-12-23

**Authors:** William A. Calo, Laura Cubillos, James Breen, Megan Hall, Krycya Flores Rojas, Rachel Mooneyham, Jennifer Schaal, Christina Yongue Hardy, Gaurav Dave, Mónica Pérez Jolles, Nacire Garcia, Daniel S. Reuland

**Affiliations:** Department of Health Policy and Management, University of North Carolina Gillings School of Global Public Health, Chapel Hill, NC USA; Cecil G. Sheps Center for Health Services Research, University of North Carolina, Chapel Hill, NC USA; Cone Health Family Medicine Residency Program, Greensboro, NC USA; Department of Health Behavior, University of North Carolina Gillings School of Global Public Health, Chapel Hill, NC USA; Center for New North Carolinians, University of North Carolina, Greensboro, NC USA; The Partnership Project, Greensboro, NC USA; Greensboro Health Disparities Collaborative, Greensboro, NC USA; Center for Health Promotion and Disease Prevention, University of North Carolina, Chapel Hill, NC USA; North Carolina Translational and Clinical Sciences Institute, University of North Carolina, Chapel Hill, NC USA; Division of General Medicine and Clinical Epidemiology, University of North Carolina, Old Clinic Bldg, 5039, Chapel Hill, NC 27599 USA

**Keywords:** Hispanic patients, Limited English proficiency, Clinic staff, Language services, Perceived discrimination, Patient misidentification, Patient satisfaction, Care coordination

## Abstract

**Background:**

Health services research of Latinos with limited English proficiency (LEP) have largely focused on studying disparities related to patient-provider communication. Less is known about their non-provider interactions such as those with patient registration systems and clinic front office staff; these interactions precede the encounter with providers and may shape how comfortable patients feel about their overall health services experience. This study explored Latino patients with LEP experiences with, and expectations for, interactions with patient registration systems and front office staff.

**Methods:**

We conducted 20 in-depth interviews with Latinos with LEP (≥18 years of age) who seek health services in the Piedmont Triad region, North Carolina. We analyzed participants’ quotes and identified themes by using a constant comparison method. This research was conducted by a community-academic partnership; partners were engaged in study design, instrument development, recruitment, data analysis, and manuscript writing.

**Results:**

Qualitative analysis allowed us to identify the following recurring themes: 1) inconsistent registration of multiple surnames may contribute to patient misidentification errors and delays in receiving health care; 2) lack of Spanish language services in front office medical settings negatively affect care coordination and satisfaction with health care; and 3) perceived discrimination generates patients’ mistrust in front office staff and discomfort with services.

**Conclusion:**

Latino patients in North Carolina experience health services barriers unique to their LEP background. Participants identified ways in which the lack of cultural and linguistic competence of front office staff negatively affect their experiences seeking health services. Healthcare organizations need to support their staff to encourage patient-centered principles.

## Background

In the United States (U.S.) over 37.6 million people speak Spanish at home, and as many as 16.5 million of these individuals report speaking English less than very well [1]. Individuals with limited English proficiency (LEP) report worse quality of health care [2], have more limited access to health care [3], and report fewer needed health care visits than their English proficient counterparts [4]. Addressing health disparities among Latinos with LEP will require health care systems to adapt not only to the health needs of this underserved population but also to their new migration patterns across the U.S. However, adapting to this changing demography has been particularly challenging in regions where rapidly growing Latino populations have been a new phenomenon [5]. In North Carolina, for example, the last two decades have been marked by unmatched increases in Latino populations across the state [6]. Over the last 10 years, the state experienced a 111 % growth rate in their Latino population, a rate that is three times higher than the national average [6]. Health care disparities among the growing Latino communities in North Carolina are well documented [7]; unfortunately, the state has substantial gaps in its capacity to fully address these disparities, including insufficient bilingual health care providers and clinic staff and culturally appropriate policies [8].

Existing health services research of Latinos with LEP have largely focused on studying disparities related to health outcomes and patient-provider communication. However, less is known about their non-provider experiences such as those with patient registration systems and their interactions with clinic front office staff (for the purpose of this of this article, these will be referred to as “front office staff”). In fact, most of the time, these interactions precede the actual encounter with health care providers, shaping how comfortable patients feel about their overall experience in getting services [9]. For instance, patients interact with front office staff to schedule and check in for appointments, ask questions about insurance coverage, provide information at each visit, and be escorted in to see health care providers [9]. Office staff also document patient language preference and collect contact information. Unfortunately, negative experiences may result from these interactions when front office staff fail to engage in culturally and linguistically competent practices. For example, Bronheim argued that patients and caregivers may experience fear of contacting doctor’s offices and clinics, feeling unwelcomed, not valued, insulted, or report being treated rudely [9]. However, we found no empirical studies that give voice to the patient perspective with regard to these non-provider issues. Since Latinos tend to receive health care in resource-constrained settings [10], a better understanding on how interactions with patient registration systems and front office staff shape their experiences seeking health services can inform the development and implementation of policies and procedures to address the cultural and linguistic capacity of staff at health agencies, clinics, and practices.

Research focusing only on interactions with health care providers may ignore important barriers in the quality of care for Latinos with LEP. To our knowledge, there has been little examination of Latinos experiences with patient registration systems and front office staff and how these interactions shape their overall experience with health care settings, particularly in areas that have seen rapid growth of Latino populations. To fill this gap, we conducted a qualitative study to explore LEP Latino patients’ experiences with, and expectations for, interactions with patient registration systems and front office staff. Since survey research does not allow individuals to express a broad range of opinions and perceptions about their experiences in seeking health care, we undertook a qualitative inquiry approach to more fully capture their experiences. Our goal in this exploratory work is to inform future community-based translational research in new destination areas for Latino migration, such as North Carolina to improve equal access to quality care for Latinos with LEP.

## Methods

### Study development and design

We developed this exploratory research under the auspices of the Clinical and Translational Sciences Institute (CTSI) at the University of North Carolina-Chapel Hill using principles of community engagement [11] in collaboration with community partners in Greensboro, North Carolina. In 2010, our CTSI Community Engagement Program established a Latino health research initiative aimed at promoting community-based translational research that engaged and was relevant to Latino communities across the state. As part of these efforts, our CTSI convened a community-academic working group in the Piedmont Triad region of North Carolina to identify health needs and priorities for Latinos, provide input and feedback critical to translational research, and help promoting enrollment in Latino health studies. This working group consisted of stakeholders from community- and faith-based organizations, the local Area Health Education Center, primary care physicians, nurses, Spanish health care interpreters, and researchers from two universities. Several partners were bilingual Latinos with interest and backgrounds working alongside Latinos with LEP through personal or professional experiences.

Through multiple discussions at working group meetings, and acknowledging a large body of research describing LEP Latino patients’ experiences with clinical providers, community partners expressed interest in exploring non-provider barriers experienced by these patients at hospitals and clinics. Specifically, the group wanted to learn more about the experiences of Latinos with LEP with patient registration systems and their interactions with front office staff, including experiences making appointments and calling for information or services, challenges registering multiple surnames in medical records, registration-related misidentification errors, availability of Spanish language services, and perceived discrimination. Several community partners expressed concerns regarding these potential non-provider barriers and shared anecdotal experiences that guided our research. For example, some observed or experienced problems related to the registration of multiple surnames, including inability of electronic health record systems to accommodate more than one surname and lack of familiarity with Latinos’ naming traditions from front office staff. In Latin American cultures, a Latino person’s full name comprises two surnames, the first one is the first surname of his/her father and the second surname is the first surname of his/her mother (mother’s maiden name). We then agreed that understanding patient perspectives and expectations, other than those of patient-provider interactions, would provide valuable information for ongoing translational research strategies aimed at improving the experiences and satisfaction of this vulnerable group of Latinos when seeking health care. In addition to identifying the topic priorities for this study, community partners were actively engaged in all stages of research including study design, instrument development, of participants, data analysis, and manuscript writing.

### Recruitment and data collection

In 2012 we conducted 20 semi-structured interviews with Latinos with LEP who seek health care services in the Piedmont Triad region. Community partners recommended using a qualitative inquiry approach for this study because conducting research in this manner provides the greatest freedom for vulnerable populations to describe a broad range of experiences and opinions, and for researchers to elicit those experiences during data collection [12]. We employed a snowball sampling strategy to recruit study participants, where eligible Latinos were identified by community partners; initial respondents were asked to refer other Latinos to the study. We relied on snowball sampling because this recruitment approach is recommended when seeking to engage and recruit hard-to-reach populations [13]. Interested individuals were eligible to participate in the study if they were Spanish-speaking Latino adults (18 years of age or older), who had received health care in North Carolina for themselves or a dependent during the previous six months, and who preferred to receive health care in Spanish language. A semi-structured interview guide was used for all interviews. The guide included questions specifically asking participants about their experiences with patient registration systems and interactions with front office staff, including patient registration processes, requests for medical appointments and care coordination, Spanish language services across practices, and perceived discrimination based on Hispanic ethnicity or LEP.

Interviews were conducted in Spanish by two bilingual research assistants, who obtained informed consent from each participant. Participants were interviewed in-person or by phone, depending on their preference. To encourage participants to freely express their opinions, we did not ask the name or location where they, or dependents, usually receive health care. Community partners advised that participants would be more likely to be candid about their experiences if opinions were elicited outside of research and clinical settings, thus all in-person interviews were held in community settings. We also administered a demographic questionnaire (e.g., age, gender, education, country of origin, and health insurance status) and a short survey to assess how frequently participants have experienced selected barriers. We used validated survey items to assess most of these variables [14, 15]. Participants received $20 as compensation for their time. The Office of Human Research Ethics at the University of North Carolina-Chapel Hill approved this research (April 4, 2012; study protocol # 12–0544).

### Data analysis

Bilingual and bicultural staff of our CTSI translated all Spanish interview field notes into English and refined translations to better reflect cultural meanings as understood by Latino Spanish-speakers. Since interview transcripts were available in Spanish and English, authors proficient in Spanish and those not proficient were able to participate in data coding and analyses. Initial analysis was conducted by four authors, including two bilingual, bicultural team members, who read the interviews and through an iterative process, identified and discussed salient themes. Subsequently, the first author read all quotes from interviews, reviewed identified themes, developed a list of codes from the domains explored on the interview guide, and began coding by using a constant comparison method, consistent with a grounded theory approach [16]. We employed established methods for solving differences in coding qualitative research by reconciling such discrepancies through group discussions and consensus [16]. Finally, descriptive statistics were calculated to summarize data from survey results.

## Results

Sample characteristics and barriers reported by study participants are shown in Table 1. Respondents were predominantly female (90 %), Mexican (90 %), and uninsured (95 %). All reported using their two surnames and speaking English less than very well. More than half (60 %) of study participants reported problems during patient registration due to confusion about their surnames. Forty percent of participants also reported that their surnames were recorded differently across health care facilities. In addition, 65 % of respondents reported experiencing difficulty at registration due to language barriers. The majority (95 %) of the sample have required assistance sometimes or always when reading materials at a doctor’s office or pharmacy. Regarding perceived discrimination during patient registration, more than half (65 %) of participants indicated that they have felt discriminated against by staff because of their Hispanic ethnicity or LEP.

**Table 1:** Summary of sample characteristics and barriers reported by study participants (*N* = 20)

	N (%) or mean (range)
*Demographics*	
Female	18 (90 %)
Age, years	35 (23–54)
Country/region of origin Mexico Central America or the Caribbean	18 (90 %) 2 (10 %)
Years in the U.S.	8 (2–17)
Education, years	9 (5–17)
Uninsured	19 (95 %)
Have two surnames	20 (100 %)
Speak English less than very well	20 (100 %)
*Barriers*	
Experienced problems during patient registration due to mishandle of surnames.	12 (60 %)
Patient names have appeared differently across various health care facilities.	8 (40 %)
Had difficulties during patient registration due to lack of Spanish language services.	13 (65 %)
Needed to have someone helping out when reading instructions, pamphlets, or other written materials at doctor’s office or pharmacy.	19 (95 %)
Felt discriminated against by patient registration staff	13 (65 %)

Analysis of participants’ interviews allowed us to identify the following recurring themes: 1) inconsistent registration of multiple surnames may contributes to patient misidentification errors and delays in receiving health care; 2) lack of Spanish language services in front office medical settings negatively affect care coordination and satisfaction with health care; and 3) perceived discrimination generates patients’ mistrust in front office staff and discomfort with services. These themes are discussed below and are accompanied by illustrative quotes. Additional quotes are shown in Table 2.

**Table 2:** Additional quotes from interviews

Theme	Sample quotes
Registration of Latino patients’ surnames	They [front office staff] didn’t have my names or last names written correctly. But they get annoyed because I don’t speak English. I told them and they got annoyed.
	I have two last names. For us, the first last name is the paternal one. But they [front office staff] put me down by my second last name, which is the maternal one. Sometimes when I call I give them the first one, but they have me down by the second one.
	They [front office staff] change my last name. They put the last one first and sometimes things get confusing.
Lack of Spanish language services	When I call to make an appointment, they’ve hung up on me like 3 times while I’m waiting to speak with the interpreter.
	Some forms are in English and I don’t understand them. You start guessing. Sometimes the Spanish forms run out.
	They [front office staff] translate the forms but they’re not right. I understand a little English, but I don’t know how to write it. I have a lot of difficulty with that. Sometimes they put the translated words backwards or wrong.
	The clinic has very little personnel that speak Spanish, so I have to wait until there’s a translator. When I call, they put me on hold for a translator and the call cuts out; I have to call again.
	When I call to make an appointment they [front office staff] don’t speak Spanish. When they don’t understand me, I try to find somebody to help me. I have my 14-year-old son who helps me quite a lot, but now that he’s out of town… I think, “What am I going to do?”
	Sometimes you go to get a blood sample checked instead of for an appointment [but] we don’t understand each other because they speak English and I don’t.
	My daughter visits a cardiologist. They [front office staff] tell me that they don’t speak Spanish there and I have to take an interpreter.
	When we go to places [clinics] where there’s nobody who speaks Spanish, I have to take my daughter, so she can translate for me.
	I have to wait longer for someone to help me, for there to be an interpreter, they have to search for an interpreter, or get one over the phone.
Perceived discrimination and mistrust	I felt discriminated because of the way they demand ID cards, like an interrogation.
	I always wonder why they ask for my ID but they don’t ask everybody, but since they speak English and I speak Spanish and I want to avoid a bad situation, I don’t say anything.
	The person that was there before, if I asked her things she got angry and answered me rudely.
	There are people that give you a nasty look, and others that act really nice. It just depends on who you get because there can always be a bad apple.
	It happened to me that one person who works doing registration was making fun of me. [He/she] had my sheet and shared it with another person. Why did [he/she] make fun of me, especially if I don’t know the language [English]?

### Theme #1: registration of patients with multiple surnames contributes to misidentification errors and delays in getting health care

Participants reported many challenges with registering their two surnames during patient registration at clinics, including difficulties locating their patient records and finding medical appointments that were registered under only one of their two surnames. One respondent commented: *“When I call to verify the appointment, they [front office staff] staff can’t find me because they register me by my second last name in some records.”* Additionally, some reported registration-associated misidentification errors because clinics’ systems failed to properly record multiple surnames. One participant said: *“Sometimes they [front office staff] make mistakes with the surnames, they gave me another person’s file, for example, and they didn’t even realize it until I noticed that the date of birth wasn’t mine.”* Participants also cited examples of incorrect order of surnames across medical facilities, which contributed to delays in obtained needed medical care; in this illustration, in getting medications: *“My mother went to the doctor and they prescribed her some medications, but when she went to the pharmacy her last names were written differently from how they are in her Medicaid. They finally gave her medications.”*

### Theme #2: lack of Spanish language services in front office medical settings negatively affect care coordination and satisfaction with health care

Respondents shared negative experiences as a consequence of not having needed Spanish language services at clinics they usually go for health care. Key challenges included the absence of medical forms in Spanish, lack of Spanish speaking front office staff, and long wait times due to limited availability of interpreters. Regarding lack of forms in Spanish, one person said: *“At the pediatrician’s office there are always forms in Spanish but at the emergency room there aren’t, they’re in English. What’s in Spanish I understand it; what’s is English I fill out what I can [understand]…”* Participants also described problems with existing Spanish forms noting that these were either translated poorly or contained unfamiliar terms. One respondent said: *“It’s very rare that there be Spanish forms available, there almost never are. Sometimes even though there are forms in Spanish, they’re not well-written and I have to ask them [front office staff] to explain the forms to me.”* Participants also experienced difficulty with making appointments over the phone or obtaining health advice because clinics did not have Spanish speaking front office staff for phone assistance. One person commented, *“Making an appointment is difficult because my wife doesn’t speak much English, so it’s hard for her to make an appointment over the phone. But if she goes to the clinic without having made an appointment, they tell her to make an appointment over the phone and she has to go back to the clinic another day.”* Others expressed frustration with long wait times at clinics because the lack of translators: *“They have a sign that says if you’ve been waiting for more than 15 min you can make a complain, but then they [front office staff] tell you that it’s because you don’t speak English and all the interpreters are busy, but citizens from here don’t have to wait like we do.”* Lack of Spanish language services also negatively affect care coordination, including getting specialty care. As one person mentioned: *“Where I usually go for health care, they have a translator but the first time I went to the ophthalmologist, they told me that they couldn’t see me because they don’t speak Spanish. They didn’t even allow me to call a friend who speaks English, so I had to make another appointment and bring him as my translator.”*

### Theme #3: perceived discrimination generates patients’ mistrust in front office staff and discomfort with clinic services

Participants’ responses uncovered perceptions of discrimination based on their Latino ethnicity, language spoken, and immigration status. One participant said*: “There are [front office] staff that treat me well and there are others that don’t… and then come discrimination, 60 % [of the time].”* Regarding perceived discrimination because of language discordance, one person commented: *“Sometimes what’s happened, more at* [name of clinic withheld] *than anywhere else, they’re very rude when I spoke to them in Spanish.”* Participants echoed similar perceptions of discrimination because front office staff apparently request identification to Latino patients but not to other individuals. As one participant expressed: *“[The front office staff asks for ID] only if you’re Hispanic. If you’re American or African-American they don’t ask for it. It’s not necessary…”* These perceived discriminatory experiences have generated patients’ mistrust in front office staff and discomfort with clinic’s services, including those services offered by translators. One person said: *“I imagine that there are things they [translators] don’t tell you…”*

## Discussion

Our exploratory research suggests that Latino patients in North Carolina experience barriers unique to their LEP background. In the present study, participants identified ways in which inappropriate patient registration systems and the lack of cultural and linguistic competence of front office staff negatively affect their experiences seeking health care, including misidentification errors and delay in getting health care due to inaccurate collection and entry of surnames, lack of needed Spanish language services, and perceived discrimination. The themes identified convey issues about the capacity of patient registration systems to register multiple surnames or to arrange timely interpreter services to Latinos with LEP, the lack of knowledge and skills of front office staff to provide information or assistance in Spanish language, and human interactions that are not respectful or culturally competent. These barriers can inhibit a health care system’s efforts to promote a safe, patient-centered environment by compromising respect for patients, contributing to errors in patient identification, limiting care coordination across clinics, and reducing satisfaction with care delivery [17].

While the frequency and consequences of Latino surnames’ registration inaccuracies are understudied in the literature, our study suggests this problem may be common among Latino patients in North Carolina. Our finding is novel because no previous studies have explored this issue of patient registration of Latino surnames and its potential effects on health care experiences. The absence of fields in electronic medical record systems and written forms to properly capture multiple surnames represents a technical barrier to respect for Latino patients’ culture, identities, and naming traditions [18]. Our findings suggests that these naming inconsistencies can result in multiple medical records for a single patient, registration-associated misidentification errors, as well as delays in health care delivery and information-sharing across medical providers. One of the most distinctive customs in Latino culture is the use of paternal and maternal surnames. As addressed by Pérez-Quiñones [19], “The problems that the two surnames present to organizations dealing with Hispanics often resides in the human and social side of the computer-human work allocation. Sure, the computer systems need to be updated to be able to handle the two surnames, but that is not a technical challenge. It is very easy to update the software needed to store and process the two surnames.” Pérez-Quiñones also noted that changing a person’s misconceptions and understanding of the Hispanic culture regarding the use of two surnames is more challenging than updating a software program [19]. Our study suggests that both technical and human barriers need to be addressed in order to properly collect Latino surnames and create a healthier atmosphere for cultural diversity in clinics serving Latinos. To achieve this atmosphere, front office staff would require organizational support to develop the knowledge, understanding and skills necessary to serve Latino patients in a manner that respects cultural and linguistic preferences. Healthcare organizations may provide ongoing training to staff in cultural competency, and incorporate cultural competency measures in individual performance evaluations [9]. For example, organizations may assess patients’ perspective on the cultural competence of front office staff with the CAHPS Cultural Competence Item Set [20] and include these into their routine staff assessments.

Participant discussions also revealed that not having appropriate language services at clinic front office negatively affect access to quality care and overall satisfaction with care. This finding builds on a small existing literature showing that language barriers among Spanish-speaking patients are associated with worse customer service from health care organizations’ staff, and lower satisfaction with care compared to their English-speaking counterparts [21–23]. For example, Moreno and Morales [24] reported that needing and not having an interpreter available for use was significantly associated with decreased rating of clinic’s staff courteousness and helpfulness among Latino patients with LEP. Ross DeCamp et al. [12] also noted that LEP Latina mothers in southwest Detroit reports large dissatisfaction with medical appointment systems and phone support because clinics lack Spanish-speaking office staff. Evidence from *Hablamos Juntos*, a national program funded by the Robert Wood Johnson Foundation to improve quality health care by providing language services to Latinos with LEP, showed that provision of trained interpreters, in contrast to no language services or use of ad hoc interpreters (e.g., family members, friends, and untrained medical or nonmedical staff), improve quality of care [24, 25]. The program developed affordable models for health care organizations to offer language access services to Latinos with LEP in communities with new and rapidly growing Latino populations [24, 25]. In terms of national policy, Title VI of the Civil Rights Law of 1964 requires recipients of Federal financial assistance to provide meaningful linguistic access to health care for patients with LEP. Under “ideal” conditions, clinics serving Latino patients with LEP in North Carolina could hire trained bilingual staff to provide a broad number of needed language services including translation of medical forms, phone assistance in Spanish, and interpreter services. However, the cost of meeting the Title VI language assistance requirements has precluded health care organizations across the country to fully implement these services [26].

Our participants also reported perceived discrimination by clinics staff, which generated mistrust in staff and discomfort with overall health services. National data shows that 30 % of Latinos believe that discrimination due to race/ethnicity is a major problem in health care settings, and 58 % of Latinos are concerned about being treated unfairly due to their ethnicity when seeking health care services [27]. Perceived discrimination is associated with inhibiting patients’ engagement with the health care system, including delays in obtaining medical care, less utilization of preventive services, and less adherence to doctor’s recommendations or treatments [28, 29]. For example, a recent study from Keller et al*.* [30] in a population of Latino immigrants in Durham County, North Carolina, showed that any perceived discrimination was associated with increased likelihood of going without needed health care (adjusted OR = 3.0, 95 % CI: 1.4-6.2). Because perceived discrimination is an access barrier to quality care, institutions should identify and address sources of perceived discrimination. Participants in our study identified clinic’s staff as a source of perceived discrimination. This finding is consistent with those of previous research conducted in other locations in North Carolina [31, 32]. Data from LATCH (Local Access to Coordinated Healthcare), a community-based program from Duke University to overcome barriers to health care access), showed that program enrollees were dissatisfied with clinic’s staff in Durham County, believing they were forced to wait longer and refused health care services by these staff based on their ethnicity [31, 32]. In order to address barriers related to perceived discrimination, organizations should establish and enforce policies and procedures to assure a non-discriminatory customer environment, promote awareness among patients about rights and grievance processes, and collect information routinely regarding patients’ race/ethnicity and monitor differential experiences with clinic’s staff [33–35].

While the findings of this qualitative inquiry are an important aspect of this study, equally noteworthy is the process by which community members and researchers collaborated throughout all stages of the project. This represented a unique effort of our CTSI to promote community-academic partnerships to support community-based translational research in North Carolina. Throughout the project, including dissemination of findings in health care organizations in the Piedmont Triad region and preparation of this manuscript, the working group provided feedback, experience and insight. In addition to help recruiting a bilingual and bicultural field interviewer, community partners advocated for recruitment and data collection methods in community settings that promoted a more secure research atmosphere, and facilitated more open discourse between participants and the interviewer.

Latinos with LEP are a particularly vulnerable subset of an already underserved ethnic community, and may be more likely to distrust health care organizations, including academic and research institutions providing health services [36]. The challenges to engaging with this population make it imperative that trust be established through natural community leaders who have an intimate understanding of the community’s strengths, needs, and concerns [37]. This project demonstrated how local partners and researchers might work together in a mutually beneficial relationship to address the needs and goals prioritized by communities. An essential component of this partnership was the shared effort to design and conduct a field study about a topic that was salient to the communities represented in the working group. Pursuing an issue prioritized by the group promoted ownership and sustained interest among members throughout the design, data collection, and dissemination stages of this research. Finally, adapting the study scope and size to the collective experiences and resources of the group contributed to the successful implementation of the project.

This study has some limitations. First, because the exploratory nature of this project, our findings should be considered preliminary. Second, the small, non-representative sample was predominantly Mexican, which limits our ability to generalize study findings to other Latino subgroups. However, Mexicans are the largest Latino subgroup in North Carolina and the U.S., and we also suspect the experiences reported in the present study are commonly faced by Latinos with LEP regardless of their country of origin. Third, almost all participants were women so men perspectives, which may be distinct, were missing from the present study. In addition, participants’ discussions were based on recalling previous experiences in health care settings, which may introduce some recall bias. Finally, although our working group included bilingual and bicultural Latinos with personal and professional ties with the Latino community in North Carolina, we did not have a Latino with LEP participating in the group or providing feedback on the present study. Future research should assess the costs of language barriers and efforts to overcome them; the scope and consequences of differential interactions between patients and clinic’s staff; and the perspectives and opinions of staff regarding their experiences serving racial/ethnic diverse populations of patients, including those with LEP.

## Conclusion

In conclusion, this exploratory study suggests the need for promotion and maintenance of an institutional culture within health care organizations that encourages patient-centered principles, such as respect, trust, and dignity, in order to improve patients’ experiences [17]. Our research pointed out to the timeliness of collecting more relevant patient-experience measures beyond patient-provider interactions as previously explored. Because of the key roles front office staff play for patients in accessing health care, organizations need to support them in the services they offer, since cultural and linguistic competence should be included at the first point of contact for a patient which is often with front office staff. We believe, as others have highlighted, that “your front office staff are the face of your practice – an expression of your philosophy, attitudes and values” [38].
